# Experimental Investigations of AlMg3 Components with Polyurethane and Graphene Oxide Nanosheets Composite Coatings, after Accelerated UV-Aging

**DOI:** 10.3390/molecules27010084

**Published:** 2021-12-23

**Authors:** Alin Constantin Murariu, Lavinia Macarie, Luminita Crisan, Nicoleta Pleşu

**Affiliations:** 1 National R & D Institute for Welding and Material Testing–ISIM Timisoara, 30 M. Viteazu Blv., 300222 Timisoara, Romania; amurariu@isim.ro; 2 “Coriolan Dragulescu” Institute of Chemistry, 24 M. Viteazu Blv., 300223 Timisoara, Romania; lmacarie@acad-icht.tm.edu.ro

**Keywords:** polyurethane coatings, graphene oxide nanosheets, AlMg3, corrosion resistance, UV ageing, EIS

## Abstract

The use of graphene (Gr) and its derivates graphene oxide (GO) showed that these materials are good candidates to enhance the properties of polyurethane (PU) coatings, especially the anticorrosion ones since graphene absorbs most of the light and provides hydrophobicity for repelling water. An important aspect of these multifunctional materials is that all these improvements can be realized even at very low filler loadings in the polymer matrix. In this work, an ultrasound cavitation technique was used for the proper dispersion of GO nanosheets (GON) in polyurethane (PU) resin to obtain a composite coating to protect the AlMg3 substrate. The addition of GON considerably improved the physical properties of coatings, as demonstrated by electrochemical impedance spectroscopy (EIS) analysis, promising improved anticorrosion performance after accelerated UV-ageing. Computational methods and Differential Scanning Calorimetry (DSC) measurements showed that GON facilitates the formation of additional bonds and stabilizes the PU structures during the ultraviolet (UV) exposure and aggressive attack of corrosive species. Limiting oxygen index (LOI) data reveal a slow burning behaviour of PU-GON coatings during UV exposure, which is better than PU alone.

## 1. Introduction

Metallic components are susceptible to corrosion in their operating environment. Corrosion is a serious threat since it affects most materials exposed to aggressive conditions. Many protection treatments could be applied to prevent/mitigate the corrosion processes (galvanisation, anodization, electroplating, and conventional coating) [1,2,3,4,5].

Organic coatings are one of the easiest and cheapest solutions, often applied on metal substrates. The limitation of coatings arises from local defects, micro cracks, or pores, which makes the coatings more or less permeable to corrosive agents (such as oxygen, water, and ions), leading to a decrease in adhesion strength and coating delamination [6,7,8,9,10,11].

Usually, there are two organic classes of coatings recognized for protective properties: epoxydic and polyurethanes or their derivatives. Limitations of epoxy coatings using are drawing by their insufficient flexibility, brittleness at low temperature, and yellow colour tendency in exterior applications over time due to degradation upon exposure to UV radiation. Polyurethane (PU) coatings have greater flexibility, adhesion, and resistance to prolonged exposure to surrounding aggressive species than epoxy coatings. 

The barrier protective properties of organic coatings can be improved by the incorporation of inorganic fillers, additives or pigments into formulations [1,2,3,4,5,6,7,8,9,10,11,12,13] (ZnO, Zn, ZrO_2_, Al_2_O_3_, SiO_2_, TiO_2_, Fe_2_O_3_, CeO_2_, Ce-H_2_O_2_, clay, boron nitride, organic phosphorus compounds, or nanoparticles of Au or Ag). Recently, graphene (Gr–planar sheet of carbon atoms, arranged in a hexagonal lattice with the thickness of one atom) and other graphene-based materials: carbon nanotubes (CNTs) and graphene oxide (GO) have found to be a helpful additive. They are able to improve the protection of metals suitable for structural components used in aerospace, automotive, and defence industries; construction of boats, oil pipelines and other metal structure equipment; and acting as a barrier for the diffusion of corrosive species (H_2_O, O_2_, Cl^−^) [14,15,16]. It has been found in [17] that in the case where the content of Gr is increased to 8 wt%, the friction coefficient of PU/Gr composite coating is 61% lower than of conventional coating.

Compared with Gr, the presence of functional groups epoxide, hydroxyl, carbonyl, and carboxyl groups on GO edge atoms [18] reduces the interplanar forces giving a hydrophilic character [19]. On the other hand, the high surface area exposed by the nanometric size particles of additives gives improved properties compared to conventional micro size [20,21,22,23]. These groups are random distributed at the edges of GO sheets. The functionalization of GO generates good sorbent in normal solid phase extraction (SPE) capable to remove different water pollutants, by electrostatic interactions and/or hydrogen bonding through these oxygen functional groups. The functionalization of magnetic GO with lauryl sulfate (MGOLS) was reported to be suitable for fast removal of dye from waste water [24,25]. 

The addition of GO in epoxy, polyacrylic, and polyurethane organic coating [26,27] has revealed that GO in a concentration of 0.1% improves the corrosion resistance of polyurethane coatings [28]. To improve the corrosion resistance several approaches for functionalization of GO are applied [29].

For example, sulfonated multiwall carbon nanotubes (SMWCNTs) are used to non-covalently modify graphene oxide (GO) to obtain modified graphene oxide (SM-GO) [30] or functionalisation of silica nanoparticle to obtain the SiO2-GO nanostructure [31], which led to the improvement of the dispersion in the coating matrix, as well as the mechanical and thermal properties of epoxy nanocomposites. In the case of PU coatings, by adding 0.1 wt.% of GO and polyisocyanate (PI) resin grafted onto the GO surface (PI-GO) nanosheets, an improvement in the corrosion protection properties was obtained [32,33]. 

Superior anticorrosion performance of waterborne polyurethane (WPU) coatings was achieved by good dispersion of dodecylbenzenesulfonic acid (DBSA)-polyaniline (PANI)/phosphorylated graphene oxide (DPPGO) [34], or by adding modified GO with polycarbodiimide (PCD) [35], or pre-dispersed reduced graphene oxide (rGO) [36].

Organic coatings are used to obtain advanced multifunctional materials with wide applications [37] such as aluminium (Al). The aluminium oxide layer [38] naturally created on the metal surface prevents the effect of oxygen and atmospheric pollutants, but this layer is unstable in corrosive environments. In Cl^-^-rich marine atmospheric environment, the higher concentration of corrosive ions penetrates the surface oxide film and easily damages it, leading to the faster dissolution of the aluminium substrate.

The aim of this work was the estimation of the influence of the UV time exposure on corrosion resistance in a saline solution of AlMg3 substrate coated with polyurethane (PU) and graphene oxide nanoparticles (GON), abbreviated as PU-GON. These investigations were performed to determine if the addition of GON in PU coatings improves the behaviour against corrosion and to understand the phenomena involved. The macroscopic colour changes during UV exposure of coatings have been analysed and correlated with the chemical changes at the molecular level. Electrochemical techniques such as polarization curves and electrochemical impedance spectroscopy EIS were used to understand the corrosion process of Al surfaces coated with PU and PU-GON coating in chloride solution (NaCl) and to study the corrosion rate of the metal surface.

## 2. Results and Discussion

### 2.1. Optical Microscopy

**Figure 1:**
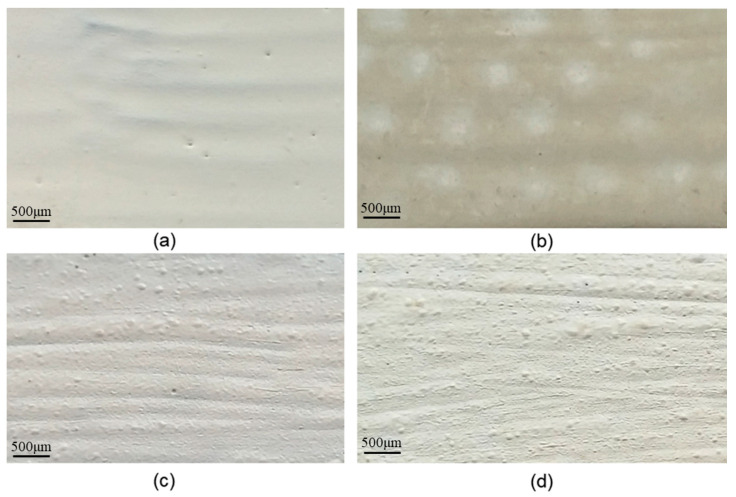
The appearance of samples surfaces: (**a**) electrode N–white grey, glossy appearance; (**b**) electrode G (aged 72 h)-yellowish-white, with degraded areas (matte), (**c**) N3 (non-aged)-dark white, semi-glossy appearance, and (**d**) G3 (aged of 72 h)-grey, matte appearance.

Figure 1a,b presents the appearance of a test sample protected with non-aged polyurethane paint compared to an artificially UV-aged test sample.

Figure 1c,d shows the appearance of a specimen protected with PU-GON, compared to the appearance of a specimen protected with the same paint, artificially aged. It is noted that, unlike the previous case presented in Figure 1, in the case of specimens protected with PU-GON, after artificial aging, apart from a change in the colour of the coating from dark white to grey, no areas with obvious degradation are highlighted.

### 2.2. Electrochemical Tests

**Figure 2:**
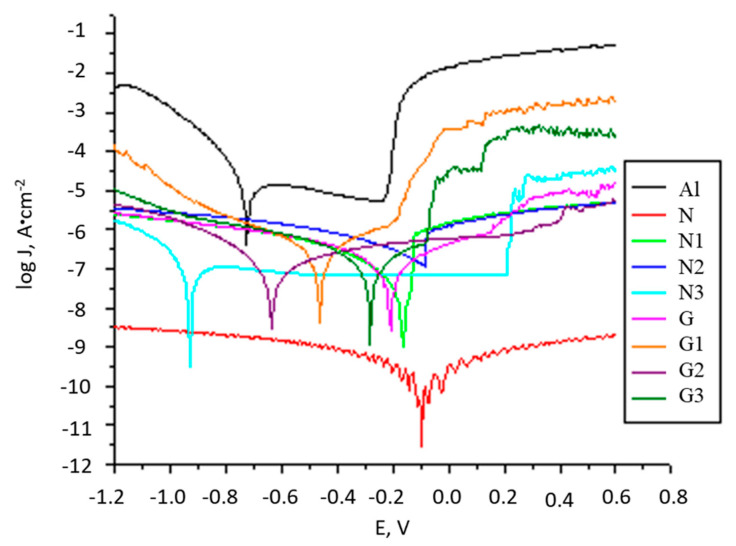
The log J versus E, for all prepared electrodes (Al, N, N1, N2, N3, G, G1, G2, and G3) immersed in 3% NaCl solution.

The passive oxide layer initially present on the AlMg3 surface has the ability to offer partial protection in a saline environment. By removing the oxide layer, by sandblasting or washing with alcohol, the surface becomes more susceptible to corrode and this is reflected in the initial value of the OCP potential (E_OCP_). During immersion in saline solution, it can be seen that for the sandblasted AlMg3 electrode (Supplementary data, Figure S1 curve a) the OCP value tends to stabilize more quickly after about 500 s at −0.295 V. For non-polished electrodes, cleaned only with distilled water (Supplementary data, Figure S1 curve b) and non-polished, degreased, and cleaned with alcohol (Figure S1 curve c), the E_OCP_ value stabilizes after approximately 1500 s at −0.250 V. The formation of aluminium oxide on the surface electrodes in the presence of dissolved oxygen in saline solution leads to an increase in the E_OCP_ value with immersion time. After one hour of immersion, the E_OCP_ stabilizes; the highest value is obtained for the unpolished surface. The E_OCP_ variation in time for all analysed electrodes (see Materials and methods, Table 3) is presented in Supplementary data, Figure S2. It is observed that the E_OCP_ moves to more positive values for almost all coated electrodes, except for the N3 electrode. The E_OCP_ value for N and G electrodes is higher compared to the AlMg3 surface (Figure S2) and indicates that, by coating, the surface becomes protected from the action of corrosive species, showing obvious corrosion inhibiting properties. Thus, although initially, the PU coating offers maximum protection, by UV exposure the corrosion protection capacity decreases dramatically (N versus N3 electrode), by the decrease in the integrity of the protective PU film and a predisposition to corrosion. The E_OCP_ values for electrodes G, G1, G2, and G3 are slightly lower but are near to the value recorded for electrodes covered with PU without GON. For G-type electrodes, through UV exposure, the integrity of the protective film is less affected. The potentiodynamic polarization curves were used to determine the values of the corrosion potential (Ecorr), corrosion currents density (Jcorr), polarization resistance (Rp), and the corrosion rates (CR) (Figure 2). All parameters represent the mean values of three measurements. The experimental data and the incertitude of parameters for Ecorr, Rp, Jcorr, and CR resulting from the interpretation of polarization curves are shown in Table S1.

The errors of experimental data are less than 8.2% and shows that all coated samples keep higher polarization resistances, a lower corrosion current density, and a lower corrosion rate than uncoated AlMg3 electrode. Electrode N, which was coated with PU, presents the lowest corrosion current density (4.199 × 10^−11^ A·cm^−2^) than the AlMg3, uncoated electrode (6.684 × 10^−5^ A·cm^−2^). The corrosion potential of electrode N shows a shift towards a more positive value, owing to the PU layer covering the metal surface. Sample G, exhibited also a lower corrosion current density (7.515 × 10^+5^ A cm^−2^) in comparison to electrodes AlMg3 and N and a more positive shift in the corrosion potential. Furthermore, the polarization resistances of these samples were two orders of magnitude higher than for uncoated surfaces and underline the advantage offered by these coatings regarding corrosion protection. For N1, N2, and N3 electrodes, Jcorr shows an increasing trend with increasing UV exposure time, follow by a decreasing trend (N2 compared to N3). The presence of GON in PU offers a significant increase in the protective capacity of the coating, highlighted by a low density of corrosion current, increased resistance to polarization, and a shift to more positive values of the corrosion potential. It seems that by UV exposure, two aspects must be present with counter effects: one is given by the post-polymerization reactions activated by UV light and the second is given by the degradation reactions of the polymeric matrix under UV radiation. In other words, the first factor ensures in time the increase of the degree of hardening, to a formation of a denser, more compact, and more protective layer. This will lead to high Rp and low Jcorr. The second factor is the degradation of the PU matrix under UV exposure that will favour the shrinking and/or formation of the cracks and pores in coatings. During the first 48 h of UV exposure, the first factor is more present and at prolonged exposure, the second one starts to become more important. For N type sample until 48 h of UV exposure (N2) a slightly increase of Rp and a decrease of Jcorr takes place as a result of an increased degree of curing of the polymer matrix. At higher exposure time (N3) the Jcorr decrease due to the shrinking and/ or formation of the cracks and pores. The presence of GON in polymer matrix brings improved corrosion behaviour: higher Rp and lower Jcorr even after 72 h of UV exposure, better values than N type electrodes. It is interesting to observe that after UV radiation, Jcorr remains almost at the same value. This suggests that GON in polymer matrix favoured the post-polymerization during UV exposure and further crosslinking reactions leading to a denser coating. This is possible due to the detachment of some groups and the generation of new active species, new crosslinking reactions to take place [39,40,41,42] (Supplementary data, Figure S3b). The colour of the protective layer changes during UV exposure and becomes grey due to the eliminating of -OH and C=O functional groups in the GON [43]. The CR values present the same trend as Jcorr. Sample N presents the lowest Rcorr (4.614 × 10^−7^ mm·year^−1^) and AlMg3 the highest value (7.344 × 10^−1^ mm·year^−1^). The Rcorr drops to lower values than AlMg3 and remains lower during UV exposure. EIS and FT-IR-ATR data confirm these results.

**Figure 3:**
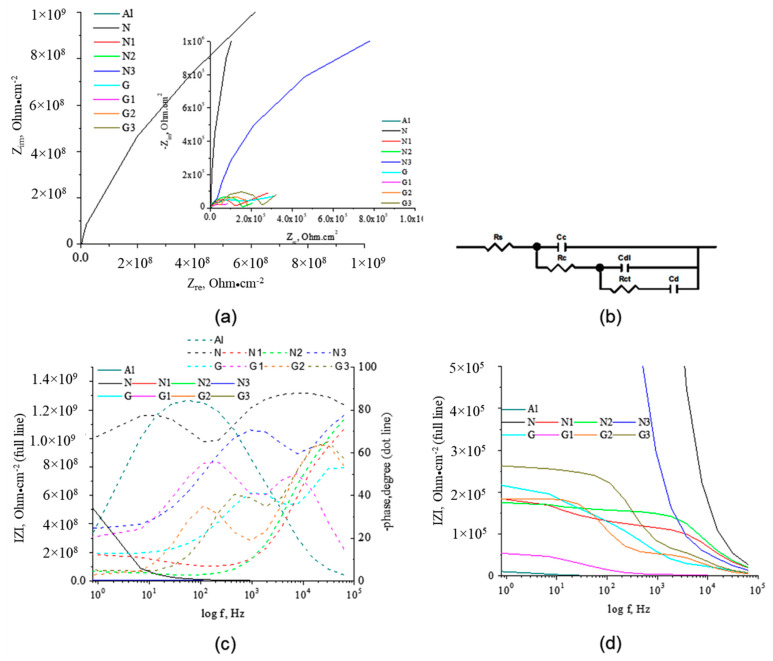
Diagrams (**a**) Nyquist and detailed Nyquist; (**b**) electric equivalent circuit; (**c**) Bode impedance modulus IZI and phase angle, and (**d**) detailed IZI versus log f, for all electrodes after 60 min immersion in 3% NaCl solution at potential OCP, V.

EIS measurement is another method in evaluation of the corrosion performance for organic coatings [44]. The Nyquist and Bode diagrams recorded for AlMg3 electrodes coated with PU immersed in 3% NaCl solution are shown in Figure 3. For a clear understanding of the capacitive loops, the Nyquist plots and Bode plots at low frequencies are detailed. For N and G samples, the Bode plots are close to a straight line with a slope of ~1 and the Nyquist plots are close to the Y-axis. Initially, the coatings act as a pure capacitor. By exposure to UV, an incomplete semicircle corresponding to a second time constant appeared, and the impedance at the low frequencies decreased. The high frequency semicircle exposes the performance of coating protection and the semicircle at low frequency was related to the products under the coating. Phase angle (theta) in the domains of high frequencies was considered a useful parameter for evaluating the protective performances of coatings [45]. Higher phase angles indicate that the current had a preference to pass through dielectric pathways, and lower phase angles show that current prefers to pass through conductive pathways in the coating. Higher theta angle shows high resistance of the coating and was observed for N and G electrodes (Figure 3b). During UV irradiation the phase angle of coating is changing as the structure of coatings is modified. The impedance spectra analysis was made using an equivalent electrical circuit (EEC) composed by a solution resistance (Rs), coating resistance (Rc), coating capacitance (Cc), double layer capacitance (Cdl), and charge transfer resistance (Rct). Cd represents the diffusion of ions from the electrolyte to the electrode interface, as modelled by a capacitance. In Table S1 are presented the incertitude of parameters Cf, Rf, Cdl, Rct, and CPEdl-T and the goodness of the fit is illustrated by the Chi-Squared parameter (the square of the standard deviation). The obtained values for Chi-Squared indicate a good correlation between the original data and the calculated spectrum and the validity of proposed EEC and low incertitude of parameters.

Constant phase element (CPE) was introduced because it better describes the actual behaviour of solid electrodes. The polarization resistance and calculated film capacity values (calculated from CPE-T value and CPE-P) are presented in Table S1. The value of perfect capacitor *C* can be calculated with Equation (1) and the impedance of constant CPE is given by Equation (2).

(1) $$Z_{CPE}=\frac{1}{T{\left(J\cdot \omega \right)}^{\varphi }}$$

where 0 < *φ* < 1 describes the deformation of the circle in the complex plane and *Q* is a constant. If *φ* = 1 CPE becomes a perfect capacitor. *ω* is the angular frequencies (in rad·s^−1^, with *ω* = 2πf), *f* is the frequency (in Hz). The *T* parameter is proportional to the capacity of the double-layer (Equation (2)):

(2) $$T=C_{ds}^{\phi }{\left(R_{s}^{-1}+A\right)}^{1-\phi }$$

$C_{ds}^{\varphi }$ = capacity of the double-layer, in F; *R_s_* = solution resistance, in Ω; *A* = electrode surface area, in cm^2^.

The IZI values are between 1.36 × 10^9^ and 1.60 × 10^5^ Ω cm^2^. The highest IZI value was obtained for the N electrode. An increase of the protective layer capacity is due to either increasing the dielectric constant or porosity of the deposited layer on the electrode or shrinking (decreasing its thickness). The lowest Cc value is present in the N sample. Cc for N type samples increases with UV exposure. This shows that with the increase of the UV exposure time, the degree of crosslinking for the PU matrix increases, and the tendency to shrinking of the film increases, which can lead to delamination. For G type electrodes, film capacity increases, most likely due to both dielectric constant and polymer film modification. For G type samples, Cc decreases with increasing UV exposure as the coating becomes denser, due to the participation of GON in crosslinking reactions, the degree of compaction of the film increases. On the other hand, the shrinking tendency of the coating is limited; GON ensures additional stabilization of the PU chains. A higher value of Cdl implies the appearance of delamination phenomena, observed in the case of N-type samples where Cdl decreases due to the shrinking of the coating during UV exposure. An increase in Rct after UV exposure indicates a decrease in porosity or an increase in the degree of crosslinking of the organic coating (UV light activates additional crosslinking processes with the generation of a compact structure). Despite the fact that at prolonged UV exposure, Rct is lower for PU GON samples, compared to PU, a beneficial aspect appears because of the establishment of additional bonds between the polymer chains. This will increase or at least stabilised the Rct. Rct increased by adding GON and by the increase exposure time to UV (Table S1). The change in layer conformation on the surface and the capacity to form a bond between water molecules and release species limit the diffusion. The exponents CPEd-P reveal the high non-homogeneity of surface in all samples. The experimental show the similar behaviour with those reported in the literature [24,27,30,31,32,33,34,46,47,48]. The G coatings investigated in this work show a higher Rct values comparatively with graphene oxide PU coatings reported by Wang and co also for Al [36]. Polymer-graphene hybrid coating, comprising two single layers of chemical vapour deposited (CVD) graphene sandwiched by three layers of polyvinyl butyral (PVB), provides complete corrosion protection to commercial aluminium alloys similar with coatings studied in this paper [49]. Other PU-graphene oxide coatings reported in the literature, similar to the G coatings reported herein, have been tested on other metal surfaces [46,47]. Studies in which the multilayer layers of graphene PU-oxide were deposited on a substrate of Cu or Fe show higher values of resistance in chloride solution than those obtained in this paper [46,47]. For nickel coated with a high quality thick multilayer graphene (MLG) in an acid solution, the reported data showed a decrease in the corrosion rate from 0.226 mm/year for pure Ni to 0.097 mm/year for Ni coated with MLG, suggesting an appropriate inhibition of G coatings [48].

### 2.3. IR-ATR Spectroscopy

IR spectra of the PU contain hard segments and soft segments of the urethane (-O-CO-NH-) or urea (-HN-CO-NH-) and segments of the polyol. The bands observed at 3450–3330 cm^−1^ were attributed to OH, NH–COO, and NH stretch, at 2924 cm^−1^ to CH_2_ stretch, at 2870 cm^−1^ to CH stretch. The bands at 1730 cm^−1^ were attributed to amide I (C=O, urethane), at 1641 cm^−1^ to amide II (C=O, urethane), at 1527 cm^−1^ and 1447 cm^−1^ to NH–COO, and NH, at 1379 cm^−1^ and 1253 cm^−1^ to CH_2_ twist, at 1072 cm^−1^ to =C–O–C and at 969 cm^−1^ to CH_3_ rocking [50,51]. The bands observed at 3450–3330 cm^−1^ give information regarding water uptake and post-polymerization processes during UV irradiation. The diffusion of water in the bulk of a coating could be monitored by the intensity of the OH vibration band. For N type coatings, an increase of water uptake with UV irradiation takes place, as the coating became more porous or delaminated from the electrode. This is supported by EIS data, the determined IZI value presents the same tendency. For G type coatings the water uptake decreases and points to the beneficial action of GON (Figure S3a). For N type samples, the intensity of the signal of the band at ~3330 cm^−1^ increases with exposure time, due to the new amide groups are formation during the post-polymerization process (Supplementary data, Figure S3a). The N-H stretching band appeared in the same domain as OH, and can give some information about modification in the hard segment of PU. It was observed that in an ordered environment N-H groups absorb in the frequency region from 3340 to 3320 cm^−1^, but the same group in a disordered environment absorbs at a frequency greater than 3340 cm^−1^ [44]. For N3, N2, G, and G1 coatings exposed to UV light the N-H stretching bands presents a tendency to absorb at a higher wavelength (marked by the arrow in Supplementary data, Figure S3a) which indicate an increase disorder degree of the coatings. The short-chain polymer chains formed initially can further participate in polymerized and/or crosslinking reactions. It is well known that as hydrogen bonding alters the distribution of electrons, hydrogen bonded groups absorb at a lower frequency than the non-bonded groups. The intensities of bands deformation of CH_2_ groups occurs at ~1450 and this band reflects the conformation of the soft segment of the cured PU made by the polyol. During UV irradiation the intensity of this band decreases, slowly for G type coatings and reveals a poorer decrease of flexibility and degradation of the coatings compared to N type coatings. Due to the presence of a C=O acceptor and a donor N-H group, the urethane linkage PU is capable of forming hydrogen bonds. For G type samples each reduced form of GON has the ability to interact via hydrogen and/or π-π bonds and interact with amide groups in the PU matrix, leading to an increased number of connections between the polymer chains. The covalent attachment of GON to PU chains via a new N–H-group, during the UV irradiation, was observed on ATR spectra by a decrease of intensity of adsorption band at ~3300 cm^−1^ and 1730 cm^−1^, which demonstrates the hydrogen bond interactions between GO and PU. Furthermore, the intensity of the C-H stretching band at 2870 cm^−1^ decreases in G type coatings, with respect to the other PUs, due to a change in the structure of the soft segment. The decrease of the ratio between heights of the absorption peak corresponding to the bond (~1641 cm^−1^, soft segments) and free carbonyl groups (1730 cm^−1^, hard segments) indicate also an improved behaviour of G samples. High values of rations of the intensities of the C-O-C bending with the respect of C=O stretching bands, indicate a higher soft segments content. The decrease of the intensities of peaks at 1412 cm^−1^ and at 1447 cm^−1^ ratio reflects alteration of the conformation of the soft segment of the PU created by the polyol component. The decrease of height intensities ratio of these peaks is a sign of a decrease in flexibility and a phase separation of hard and soft segment domains and is observed mostly for the N type of sample. The UV exposure can generate free radicals. Hydrogen from the methylene (-CH_2_-) groups leave and generate in polymer a peroxy radical (PU-O2•) and polymer hydroperoxide (PU-OOH). UV-induced cleavage of PU-OOH leads to the formation of alkyl radicals (formation of a polymer oxy radical (PU-O•) radical, or hydroxyl group (PU-OH)). These radicals cause crosslinking [52].

**Figure 4:**
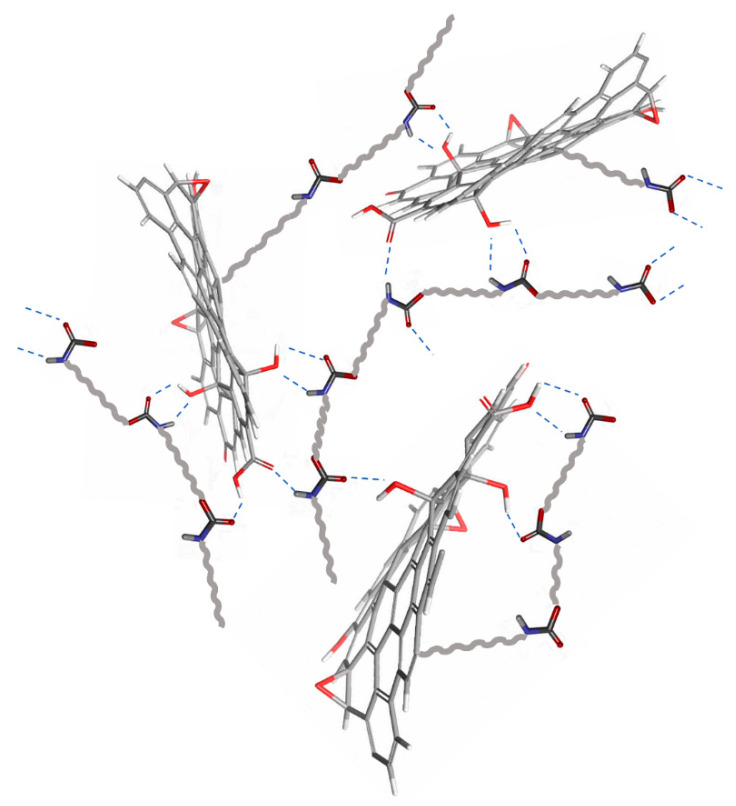
Schematic representation of possible interaction of reduced GON with PU under UV irradiation.

The bonding realized by the PU with reduced GON forms can be regarded as analogous cross-linker. GON provide cross-linking points in the system and offer and healing to the coatings (Figure 4) [53].

### 2.4. Computational Results

**Figure 5:**
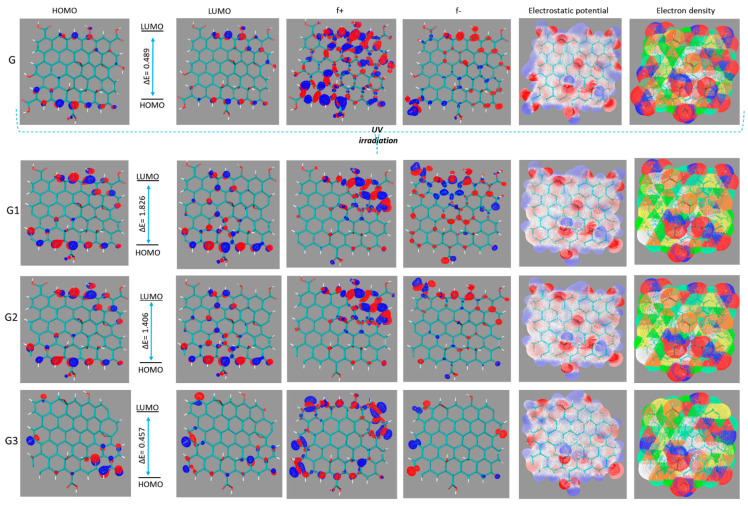
HOMO, LUMO orbitals, Fukui, electrostatic potential, and electronic density map of GON (G) and reduced GON forms (G1, G2, and G3).

To further understand the mechanism of GON action, we propose a series of molecular models, taking into account that by reduction the number of functional groups on GON is changing. The 5 × 5 graphene models (Figures S3 and S4) with different number of –OH (hydroxyl), -COC- (epoxide ring), –COOH (carboxyl), and > C=O (carbonyl) was constructed. The functional groups were designated at both the GON edges and the plane perpendicular to it and the possible forms of reduced GON which can be formed by UV exposure are portrayed in Figure 5. With the increase in UV exposure the number of carboxyl, hydroxyl, and epoxy groups present on GON decrease, and an unsaturation appear as is presented in Figure 5, Figures S3b and S4. In the reduction of GO, the hydroxyl, carboxyl, and epoxy groups (-COC-) could be removed easily, but the carbonyl groups (C=O) could hardly be removed [54]. For this reason, in our computational work at higher UV irradiation time, the carbonyl groups remained in reduced GON forms.

The accuracy of the B3LYP/6-31G(d,p) method was tested by comparison the experimental and theoretical bond length. The values of bond lengths (1.22 Å (–C=O), 1.41 Å (–C=C-), and 1.36 Å (–C-O(H)) for the optimized GOx is in good accordance with the found experimental ones [55]. Additionally, the absence of any negative frequencies insured the true energies minima of the GONs. The calculated electronic properties of the optimized GON and GON reduce forms and are shown in Table 1. It is well known that the HOMO energy shows the propensity of molecules to donate electrons, while the LUMO energy shows the propensity of molecules to accept electrons, and therefore to be adsorbed on the metal surface. The negative values for E_HOMO_, and E_LUMO_, indicate a charge transfer with a metal surface, and the absorption process may take place. The results reveal that the order of increasing E_HOMO_ of the reduced GON forms present in coatings is G1 < G3 < G2. This indicates that reduced GON forms present in coatings G1 has the least tendency to donate electrons to the metal surfaces, while reduced GON form present in coatings G2 has the highest tendency to donate electrons to the appropriate vacant orbitals. The lowest value of E_LUMO_ for G3 indicates that this molecule would accept electrons. The results obtained for ionization potential and for electron affinity show that the inhibition efficiency of GONs increases with increasing ionization energy but decreases with decreasing electron affinity. This is obvious because ionization potential is directly associated with E_HOMO_ and electron affinity to the E_LUMO_. The efficiency of the inhibition is consequently similar to that obtained for the E_HOMO_ and E_LUMO_ results. The low value of the energy gap (∆E) for reduced GON form in coating G3 compared to G1 and G2 shows that it absorbs quickly because a small gap involves less excitation energy to take out electrons from the last occupied orbital. This is a preliminary prediction that G3 has higher corrosion inhibition efficiency. A similar tendency was observed concerning the chemical hardness values. Information on the anticorrosive power of reduced GONs obtained from the E_HOMO_ and E_LUMO_ results is in line with that obtained from the Fukui functions (Figure 5). A molecule with a low value, GON form in coating G3 (here), indicates that this form can interact more easily not with the surface but also with PU chains (due to the double bond capable to bind to free PU radicals generated in the system during irradiation) [56,57,58,59,60,61]. These radicals capture and lead to a healing of coating and finally to better anti-corrosion behaviour. This is confirmed by the presence of Fukui orbitals in the region of free radicals of GONs (especially for G3). The favourite sites for the nucleophilic attack are the atoms in the GONs where the presence of Fukui orbitals (f+) was observed (Figure 5). These sites are to some extent associated with the LUMO orbitals and measure the reactivity towards a donor compound. The preferred sites for the electrophilic attack are highlighted by the presence of Fukui orbitals (f−), and are to some extent related with the HOMO orbitals and estimate reactivity toward an acceptor compound (Figure 5).

Each reduced form of GON has the ability to interact via π-π bonds and interact with amide groups in the polymer matrix, leading to a large number of connections between the PU chains. The covalent attachment of PU onto the GON via the amide linkage was confirmed by FT-ATR. The C=O stretching mode of the carboxyl group on GON, which is supposed to appear at 1732 cm^−1^, is present as a new vibration at about 1689 cm^−1^ is assigned to the C=O mode of the amide group. The electronegativity values are within the range 4.09–4.72, which suggests that all the reduced GON forms have great potential of relocating an electron to the low-lying vacant orbital of the metal. The electrostatic potential mapped on the electron density surface of each reduced form is displayed in Figure 5. The asymmetric charge distribution (negative charge in red) on the reduced GON forms denotes that each studied systems have reactive adsorption sites for bonding. Theoretical simulation is useful for establishing a strategy for exploring GON structures before experimental measurements, which leads to lower corrosion costs. The quantum chemical results are well correlated with all experimental observations.

### 2.5. Thermal Analysis

The TG curves show the appearance of two major mass loss stages (Supplementary data, Figure S5). PU undergoes thermal degradation due to the dissociation of the urethane linkage, breaking of the PU chain, and urethane bond [62]. The first stage involving mass loss occurs in the temperature range of 90–150 °C is due to the loss of water or residual solvent in the sample, but also the breaking of the short-distance connections of the rigid segments of PU. In the temperature range 220–400 °C, a second stage of massive mass loss associated with the decomposition of the PU chain takes place. The decomposition of the PU chain implicates two maximum decomposition rates at 325 °C and 380 °C. The DSC curves shown in Figure S6 illustrate the endothermic processes that take place at the heating of the samples at 25−350 °C, followed by a cooling process and then another heating cycle at 25−350 °C. After the cooling process on the curves of the 2nd heating process, the absence of endothermic processes is a result of PU degradation in the first heating cycle (process starting at ~240 °C). In the first heating the first endothermic process appears in the temperature range of 45−75 °C, and is attributed to the breakage of short-distance linkages of the hard segments of the PU, the bonds there are formed during the curing process that takes place at ambient temperature. The endothermic peak in the temperature range of 80–150 °C corresponds to the rupture of the long-distance linkages of the hard segments of the PU chain. The endothermic peak around the temperature range of 175−290 °C corresponds to both the breakage of H-bonds between the PU chain segments and the decomposition of the structures (biuret, allophane, and urethane type). The feature of DSC curves is similar for all samples; the endothermic effects are not proportional to the amount of sample taken in the analysis. The increase in UV exposure time for these samples shows a decrease in extrapolated temperatures associated with endothermic processes, because of PU rearrangement in coatings and interaction of PU with reduced GON forms in G type coatings. 

### 2.6. Limiting Oxygen (LOI) Index

Limiting oxygen index (LOI) measurements is a precision method for determining the relative flammability of various materials. The minimum concentration of oxygen required to support combustion was determined on the powdered sample according to modified ASTM D2863-70. The LOI values (Supplementary data, Figure S7) are in the range 24−27% close to other investigated polymeric systems [63]. For G type samples a decrease of the heat quantity associated with the endothermic peaks in the temperature range of 80−150 °C and, respectively, 175−290 °C appears as a result of the formation of additional short and long-distance links between the PU segments. This indicates that GON does not change the thermal decomposition profile of the sample but is capable of decreasing the rates of mass loss and undergoing an endothermic decomposition in the range of temperatures at which combustion takes place. This endothermic reaction helps to withdraw heat from the substrate [64]. Higher heat resistance of rigid polyurethane foam with GO (FRPU/fGO) specimen was reported to present also a synergistic effect between fGO and EG/DMMP [65]. The thermal analyses confirm the beneficial role of GON in increasing the resistance to UV exposure of the PU coating while maintaining the corrosive protection capacity and slowing down the mass loss.

## 3. Materials and Methods

### 3.1. Material

#### 3.1.1. Electrodes

A 2 mm thick plate of AlMg3 (EN AW 5754) was used as substrate in the experimental study. The chemical composition of the substrate is presented in Table 2.

The passive oxide layer of the AlMg3 substrate prevents exposure to air pollutants and oxygen but is unstable in corrosive environments. Thus, to achieve additional protection of the AlMg3 substrate, Purmal S-70 polyurethane resin (Manufacturer: Malchem Sp. z o.o., Sułkowice, Poland) and GON in a concentration of 4 mg/mL, in a proportion of 1%wt in the polymer matrix, was used in the experimental program.

#### 3.1.2. Polyurethane Resin and GON

Purmal S-70 PU paint used in the experiments is a direct to metal (DTM) 2-in-1 two-component PU paint, resistant to mechanical action, UV radiation, and chemical agents, with anti-corrosive properties, which can be applied in thick layers, has a semi-glossy appearance, and RAL 9010 MIX colour. The paint uses a special hardener PUR 603, the resulting mixture must be applied within a maximum of 8 h.

**Figure 6:**
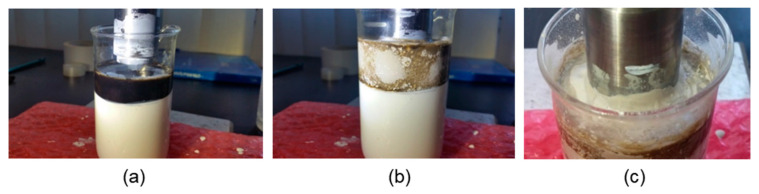
Ultrasound processing steps: (**a**) initial stage; (**b**) processing; (**c**) detail.

Source Graphene, Romania (www.sourcegraphene.com), supplied GON suspension in water. GON has high hydrophilicity, is dark brown and odourless. The thickness of GON used starts from 0.9 nm and can reach up to several nanometres; the maximum size is less than 7 µm. Ultrasound cavitation technique was used for surface modification of GON to achieve a proper dispersion [66] in PU resin. Ultrasonic processing was performed at 36.24 kHz. Figure 6 presents the ultrasonic processing steps to obtain the nanocoating PU-GON.

The graphite oxide suspension in water was sonicated for four hours to obtain a homogeneous dispersion of GON in the PU matrix. The electrodes of Ø12 mm were cut off from the AlMg3 sheet of 2 mm. Before each determination, the surfaces of electrodes were sandblasted and before coating, the surfaces of the electrodes are cleaned with ethanol and acetone and then dried in air. The films were applied by brush on AlMg3 substrate. After the deposition, all coatings were air-dried for 24 h at ambient temperature. The thickness of coatings was 120 ± 5 μm. Labels of electrodes covered with DTM Purmal S-70 PU and with DTM Purmal S-70 PU-GON start with N (Table 3). Further, the electrode sets were UV-aged by exposure for 24 h, 48 h, and 72 h to UV light. For these electrodes, the labels start with G (Table 3).

### 3.2. Methods

#### 3.2.1. Accelerated Aging Test with UV 

**Figure 7:**
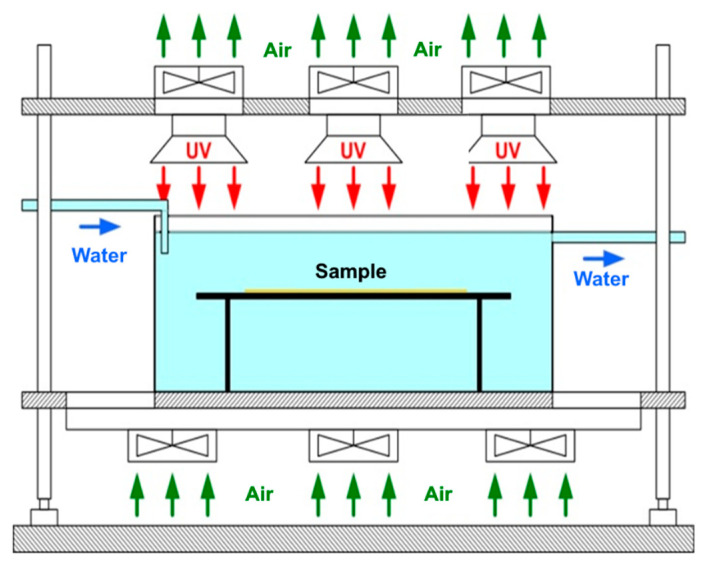
The principled scheme of the installation for accelerated UV-aging.

To assess the behaviour over time of coatings, an accelerated aging process with ultraviolet radiation was performed by exposure to different durations. The principled scheme of the installation for accelerated aging with UV radiation is presented in Figure 7.

Aging setup was developed by the authors according to the Patent number: RO131897-A2. The system is equipped with a microprocessor that allows the control and monitoring of the process by using of a control panel with touch screen, thermocouples for measuring water and indoor temperatures. The aging system is equipped with 6 Philips CLEO HPA 400/30 SD UNP lamps of 400 W. UV lamps emit ozone-free radiation, mainly in the UVA range with a wavelength between 300 and 400 nm. During the experiments, the water temperature was set at 23 ± 2 °C, the distance between lamps was 30 mm, and the distance between the sample and the lamps was 300 mm. Thus, the obtained intensity of UV radiation in the positioning area of the sample was approximately 45 mW/cm^2^ [67].

The electrochemical measurements (polarization curves, impedance spectroscopy EIS, FT-IR-ATR spectroscopy, and thermal methods TG and DSC) were performed.

#### 3.2.2. Electrochemical Tests

Electrochemical impedance spectroscopy (EIS) experiments were conducted at room temperature in a conventional one-compartment three-electrode cell, equipped with two graphite counter electrodes and a silver-silver chloride (Ag/AgCl) reference electrode with a potentiostat/galvanostat Autolab 302N and FRA2 impedance module. Before EIS measurement, the coated electrodes were initially kept in the supporting electrolyte (aqueous solution containing 3% NaCl) at an open circuit potential (OCP) for 1 h until a stable state was attained. The measurements were recorded in the frequency range 0.1 Hz to 100 kHz, at the sinusoidal potential amplitude of 10 mV, at open circuit potentials [9,44]. The collected data were fitted to the equivalent electrical circuit by a complex non-linear least squares’ procedure using the ZView-Scribner Associated Inc., software (Zwiev, Connecticut, NC, USA). The polarization curves were recorded in order to determine the corrosion rate of AlMg3 coated samples. All tests were performed three times to obtain good reproducible results.

#### 3.2.3. IR-ATR Spectroscopy

Spectroscopy in IR-ATR was used to identify structural changes in the protective organic film by UV exposure, but also by immersion in saline solution. The FT-IR spectrum was recorded in the range 4000−400 cm^−^^1^ on a JASCO−FT/IR-4200 spectrometer ATR (MIRacle) (ATR, JASCO Corporation, Tokyo, Japan).

#### 3.2.4. Computational Methods

The graphite oxide GON has been modelled and optimized using density functional theory (DFT) with the Becke, three-parameter, Lee–Yang–Parr (B3LYP) functional and, the 6-31G(d,p) basis set level of theory, using the Jaguar module of the Schrödinger package [68]. These quantum chemical methods are extensively used to study physicochemical properties and to predict the adsorption centres of the GON responsible for the interaction with the metal surface [69,70,71,72]. In addition to the E_HOMO_ (energy of the highest occupied molecular orbital) and the E_LUMO_ ( energy of the lowest unoccupied molecular orbital) values, the DFT simulations make it possible to estimate other parameters, using Koopman’s theorem [73,74], such as: the energy gap (ΔE = E_LUMO_ − E_HOMO_), the electron affinity (A = −E_LUMO_), the ionization potential (I = −E_HOMO_), the electronic hardness of the molecule (η = (I − A)/2), the global softness of the molecule (σ = 1/2η), the electronegativity of the molecule (χ = (I + A)/2), and the chemical potential of the molecule (μ = −(I + A)/2). These descriptors allow the correlation between the adsorption capacity of the GON molecules and their tendency to donate/accept electrons. Furthermore, in order to describe the local reactivity and chemical behaviour of different sites in GON molecules, the Fukui functions (f(k)) were calculated. The most susceptible sites for nucleophilic and electrophilic attacks were designed, and are represented by the f+ and f−, respectively [75].

#### 3.2.5. Thermal Analysis

Thermal analysis was recorded in nitrogen with TG/DTA/DSC Mettler Toledo (Mettler-Toledo AG, Analytical, Schwerzenbach, Switzerland), on AlMg3 pan also tracks changes in thermal behaviour from exposure to UV radiation and saline. The determinations were performed in a nitrogen atmosphere with a heating rate of 10 °C/min.

#### 3.2.6. Limiting Oxygen (LOI) Index

LOI was determined by Limiting Oxygen Index Chamber 340AJH0038 (LOI Dynisco, Franklin, MD, USA), according to modified ASTM D2863-70. The samples are of 6.5 mm width, 70 mm length, and 100 µm thickness. The minimum concentration of oxygen in a mixture of oxygen and nitrogen capable to support a 3 min burn is measured. LOI values represent the mean value of three tests.

#### 3.2.7. Optical Microscopy 

The surface morphologies of the treated samples were investigated by the optical Microscope Zeiss Stemi 508 (Zeiss Stemi, Carl Zeiss Microscopy GmbH, Jena, Germany).

## 4. Conclusions

The samples subject to UV rays are yellowing and the gloss of the film diminishes as oxygen content increase in the coatings. The addition of GON considerably improved the physical properties of coatings, as demonstrated by EIS analysis, promising improved corrosion performance after accelerated age by UV exposure. DSC measurements showed that GON facilitates the formation of additional bonds and stabilizes the PU structures during the UV exposure and to aggressive attack of corrosive species. By exposure to UV, graphene oxide is reduced, which brings a major colour change to the paint. All coated samples possess higher polarization resistances, a lower corrosion currents density, and lower corrosion rates than uncoated AlMg3 electrodes. By incorporation of GON into PU, improved corrosion behaviour was achieved: higher Rp and lower Jcorr even after 72 h of UV exposure, better values than polyurethane matrix alone. The cleavage of the urethane linkages takes place during the UV irradiation with the generation of new free species (free radicals). Two aspects with counter effect are observed during UV exposure: post-polymerization and degradation reactions of the polymeric matrix. The decrease of the intensities of peaks associated with the alteration of the conformation of the soft segment of the indicates a phase separation of hard and soft segment domains observed mostly for coating polyurethane matrix without graphite oxide. The corrosion rate (Rcorr) in 3% NaCl is high for the uncoated AlMg3 electrode, the corrosion rate drops substantially after coating. As GON participates in crosslinking reactions, the degree of compaction of the film increases. The shrinking tendency of the coating is limited by GON which ensures additional stabilization of the PU chains. For N samples, an increase of the extrapolated temperatures for each of the peaks associated with endothermic processes is observed with the increase in UV exposure time. For G samples a decrease in the amount of heat associated with endothermic peaks in the temperature range of 80–150 °C and 175–290 °C was observed by addition with graphs and by increasing the exposure time at UV, as a result of the formation of additional short and long-distance links between the hard segments of the PU. The LOI data reveal a slow burning behaviour of PU-GON coatings during UV exposure, better than PU alone. Furthermore, the estimated quantum chemical descriptors with DFT calculation give key information about the electronic structure, conformation, and reactivity of reduced GON compounds, and support their anticorrosive action.

## Figures and Tables

**Table 1:** The predicted electronic properties of the GON forms.

		G	G1	G2	G3
E_LUMO_	(eV)	−3.8494	−3.3281	−3.5322	−4.4937
E_HOMO_		−4.3388	−5.1540	−4.9387	−4.9503
Energy gap, ∆E		0.4895	1.8259	1.4064	0.4566
Electron affinity, A		3.8494	3.3281	3.5322	4.4937
Ionization potential, I		4.3388	5.1540	4.9387	4.9503
Chemical hardness, η		0.2447	0.9130	0.7032	0.2283
Chemical potential, μ		−4.0941	−4.2410	−4.2354	−4.7220
Softness, σ		2.0431	0.5477	0.7110	2.1902
Electronegativity, χ		4.0941	4.2410	4.2354	4.7220

**Table 2:** Chemical composition of AlMg3 (EN-AW-5754) (%).

Grade	Si	Fe	Cu	Mn	Mg	Cr	Zn	Ti	Other	Al
5754	0.4	0.4	0.10	0.1–0.6	2.6–3.6	0.3	0.15	0.15	0.05	Rest

**Table 3:** List of prepared and analysed samples.

Nr. Crt.	Label	PU	GON, %	Exposure Time to UV Light, Hours	OCP (V)
1.	Al	0	0	0	−0.239 ± 0.026
2.	N	Purmal S-70	0	0	0.101 ± 0.053
3.	N1	Purmal S-70	0	24	−0.248 ± 0.017
4.	N2	Purmal S-70	0	48	−0.253 ± 0.042
5.	N3	Purmal S-70	0	72	−0.461 ± 0.063
6.	G	Purmal S-70	0.3	0	−0.175 ± 0.006
7.	G1	Purmal S-70	0.3	24	−0.261 ± 0.028
8.	G2	Purmal S-70	0.3	48	−0.196 ± 0.011
9.	G3	Purmal S-70	0.3	72	−0.131 ± 0.006

## Data Availability

The data that support the results and findings of this study is available from the corresponding author upon request.

## Supplementary Materials

The following are available: Figure S1: The time variation of EOCP in V, for different AlMg3 electrodes immersed in 3% NaCl solution: (a) sandblasted wash with distilled water, (b) non-polished electrode clean with distilled water, and (c) non-polished electrode clean with alcohol, Figure S2: The time variation of EOCP in V, for all prepared electrodes (Al, N, N1, N2, G, G1, G2, and G3) immersed in 3% NaCl solution, Table S1: Corrosion potential (Ecorr), polarization resistance (Rp), corrosion current density (Jcorr), and corrosion rate (CR) values obtained from polarization curve and coating capacity and polarization resistance from EIS data for the electrodes, Figure S3: (a) FTIR-ATR spectra for Al, N, N1, N2, G, G1, G2, and G3 samples and (b) schematic action of UV light upon PU-GON coatings, Figure S4. Reduction of GON under UV irradiation (modelled by removing of functional groups marked by circles), Figure S5: The TG in a nitrogen atmosphere with a heating rate of 10 °C/min curves for Al, N, N1, N2, G, G1, G2, and G3 samples, Figure S6: DSC curves for (a) N, N1, N2, and N3 and (b) G, G1, G2, and G3 samples in nitrogen, Figure S7: LOI data.

## Sample Availability

Samples of the compounds are not available from the authors.
