# Supplementary material for: Experimental Investigations of AlMg3 Components with Polyurethane and Graphene Oxide Nanosheets Composite Coatings, after Accelerated UV-Aging
Source: Molecules. 2021 Dec 23;27(1):84. doi: 10.3390/molecules27010084 (PMC8746964; doi:10.3390/molecules27010084)
Supplement: Supplementary file 1 [file molecules-27-00084-s001.zip › molecules-1514607-supplementary.pdf]

molecules

MDPI

Supplemenatry Materials

Experimental investigations of AlMg3 components with polyurethane and graphene oxide nanosheets composite coatings, after accelerated UV-aging

Alin Constantin Murariu [1], Lavinia Macarie [2], Luminita Crisan [2*] and Nicoleta Pleşu [2*]

[1]National R&D Institute for Welding and Material Testing – ISIM Timisoara, 30 M. Viteazu Blv. 300222, Timisoara, Romania; amurariu@isim.ro (A.C.M)

[2]"Coriolan Dragulescu" Institute of Chemistry, 24 M. Viteazu Blv. 300223 Timisoara, Romania; lmacarie@acad-icht.tm.edu.ro (L.M.); lumi_crisan@acad-icht.tm.edu.ro (L.C.); plesu_nicole@yahoo.com (P.N.)

* Correspondence: lumi_crisan@acad-icht.tm.edu.ro; plesu_nicole@yahoo.com

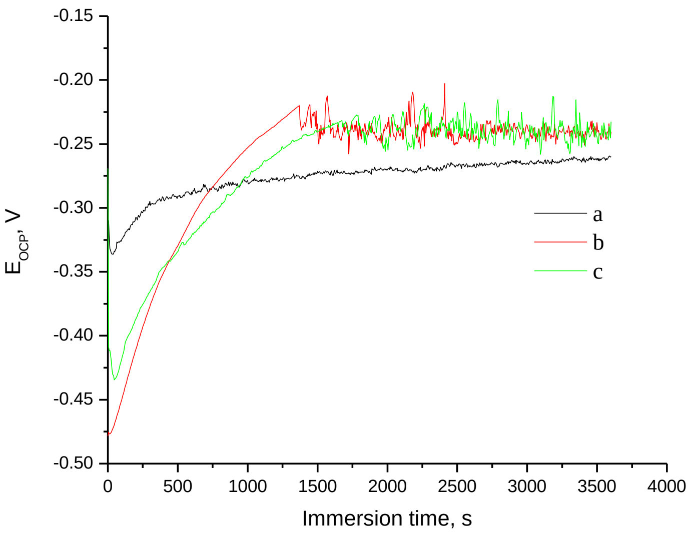

Figure S1. The time variation of EOCP in V, for different AlMg3 electrodes immersed in 3% NaCl solution: a) sandblasted wash with distilled water, b) non-polished electrode clean with distilled water, and c) non-polished electrode clean with alcohol

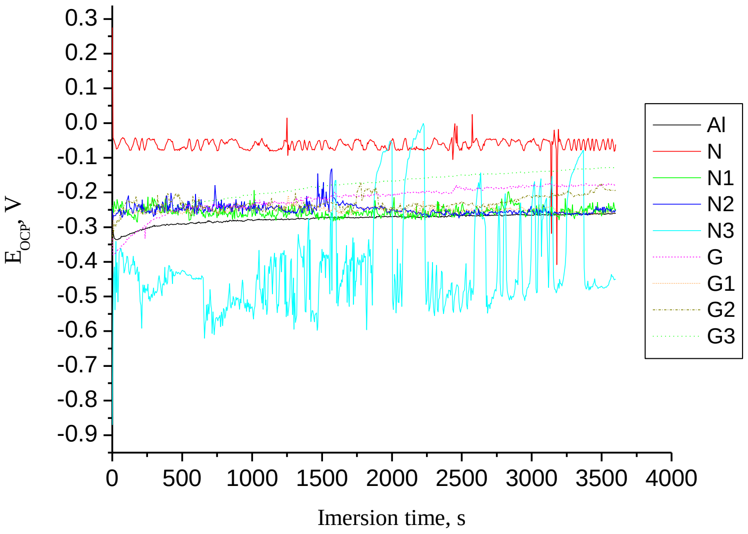

Figure S2. The time variation of EOCP in V, for all prepared electrodes (Al, N, N1, N2, G, G1, G2, G3) immersed in 3% NaCl solution.

Table S1. Corrosion potential (Ecorr), polarization resistance (Rp), corrosion current density (Jcorr), and corrosion rate (CR) values obtained from polarization curve and coating capacity and polarization resistance from EIS data for the electrodes.

**Substrate**	**Polarization curve**				**EIS**						
	**Ecorr, obs, V**	**Rp, Ohm**	**Jcorr, A.cm^{-2}**	**CR, mm·year^{-1}**	**Chi-Sqr**	**C$_f$, F.cm^{-2}**	**R$_f$, Ω·cm^2**	**C$_{dl}$, F.cm^{-2}**	**R$_{ct}$ Ω·cm^2**	**CPE$_d$T(+), F.cm^{-2}.s^{-1} ϕ**	**CPE$_d$-P,ϕ**
Al	-0.726 ±0.011	2.231x10^{+3} ±0.028	6.684x10^{-5} ±2.9x10^{-6}	7.344x10^{-1} ±0.002	5.33x10^{-3}	3.4 x10^{-4} ±0.1x10^{-5}	2.07x10^{+3} ±2.3x10^{2}	-	-	-	-
N	-0.044 ±0.009	1.323x10^{+9} ±0.031	4.199x10^{-11} ±9.1x10^{-13}	4.614x10^{-7} ±5 x10^{-9}	6.19x10^{-4}	9.40 x10^{-11} ±5 x10^{-13}	98.62 ±23.6	1.34x10^{-10} ±2.9x10^{-11}	1.36x10^{+9} ±2.3x10^{+8}	4.81x10^{-10} ±1.7x10^{-11}	0.46 ±0.01
N1	-0.166 ±0.004	3.623x10^{+5} ±0.072	2.882x10^{-7} ±8.6x10^{-9}	3.167x10^{-3} ±7x10^{-5}	7.21x10^{-3}	1.14 x10^{-10} ±7.3x10^{-11}	5.23x10^{+4} ±5.1x10^{+3}	1.74x10^{-10} ±2.6x10^{-11}	1.88x10^{+5} ±5.1x10^{+3}	4.64x10^{-6} ±5.1x10^{-8}	0.30 ±0.01
N2	-0.940 ±0.010	3.088x10^{+5} ±5.130	7.338x10^{-7} ±1.4x10^{-8}	8.063x10^{-3} ±2x10^{-5}	8.19x10^{-4}	1.22 x10^{-10} ±1.3x10^{-12}	8.85 x10^{+4} ±8.3x10^{+3}	2.90x10^{-10} ±3.2x10^{-11}	1.60x10^{+5} ±8.3x10^{+3}	1.35x10^{-5} ±5.1x10^{-6}	0.29 ±0.01
N3	-0.931 ±0.009	1.684x10^{+5} ±4.054	9.182x10^{-7} ±9.4x10^{-9}	1.299x10^{-3} ±3x10^{-5}	2.63x10^{-2}	1.89 x10^{-10} ±1.0x10^{-13}	8.26 x10^{+4} ±5.7x10^{+3}	3.64x10^{-10} ±0.6x10^{-11}	7.54x10^{+5} ±8.3x10^{+3}	9.80x10^{-8} ±2.6x10^{-9}	0.24 ±0.03
G	-0.203 ±0.009	7.515x10^{+5} ±0.403	2.12x10^{-7} ±1.3x10^{-8}	2.330x10^{-3} ±9x10^{-6}	2.69x10^{-4}	4.15 x10^{-10} ±1.0x10^{-13}	2.56 x10^{+4} ±2.5x10^{+3}	2.73x10^{-9} ±8.7x10^{-11}	1.68x10^{+5} ±6.4x10^{+3}	3.32x10^{-6} ±3.9x10^{-8}	0.15 ±0.01
G1	-0.463 ±0.005	1.589x10^{+5} ±1.433	2.625x10^{-9} ±6.9x10^{-10}	2.884x10^{-3} ±3x10^{-5}	8.07x10^{-4}	1.81 x10^{-8} ±0.9x10^{-10}	3.45 x10^{+3} ±6.4x10^{+2}	8.06x10^{-8} ±5.2x10^{-10}	5.38x10^{+4} ±6.4x10^{+3}	1.11x10^{-5} ±9.2x10^{-7}	0.18 ±0.02
G2	-0.636 ±0.007	3.311x10^{+5} ±3.537	2.017x10^{-7} ±3.3x10^{-9}	2.216x10^{-3} ±3x10^{-5}	7.51x10-4	5.94 x10^{-10} ±1.9x10^{-11}	5.15 x10^{+4} ±5.4x10^{+2}	1.86x10^{-8} ±5.1x10^{-9}	1.14x10^{+5} ±1.9x10^{+3}	1.28x10^{-5} ±1.4x10^{-7}	0.95 ±0.01
G3	-0.282 ±0.002	3.815x10^{+5} ±3.418	1.174x10^{-7} ±7.2x10^{-9}	2.089x10^{-3} ±8x10^{-6}	8.11x10^{-4}	4.19 x10^{-10} ±2.1x10^{-12}	6.64 x10^{+4} ±6.1x10^{+2}	3.37x10^{-9} ±8.3x10^{-10}	2.20x10^{+5} ±5.7x10^{+3}	7.76x10^{-6} ±3.4x10^{-11}	0.50 ±0.03

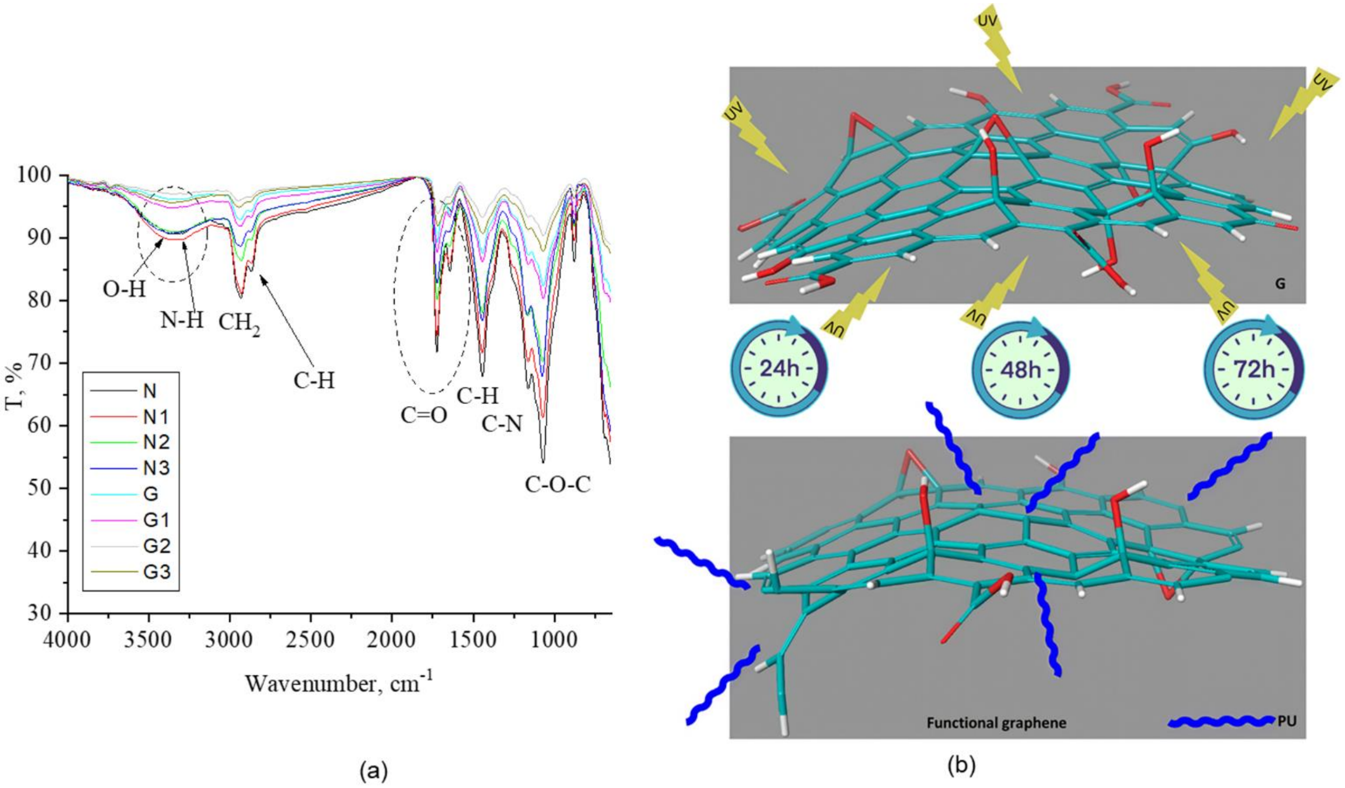

Figure S3.a) FTIR-ATR spectra for Al, N, N1, N2, G, G1, G2, G3 samples and b) Schematic action of UV light upon PU-GON coatings

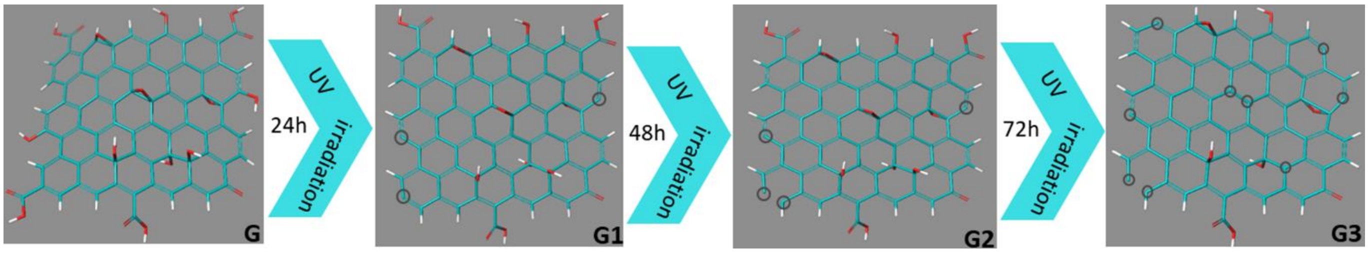

Figure S4. Reduction of GON under UV irradiation (modelled by removing functional groups marked by circles)

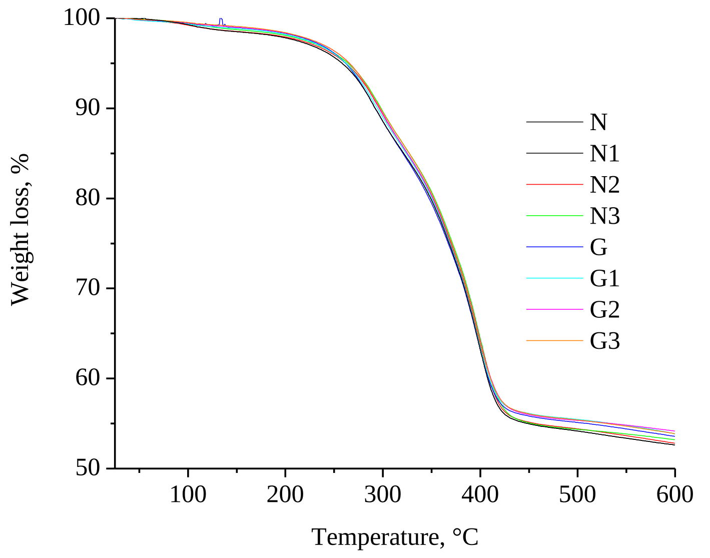

Figure S5. The TG in a nitrogen atmosphere with a heating rate of 10 ° C / min curves for Al, N, N1, N2, G, G1, G2, G3 samples

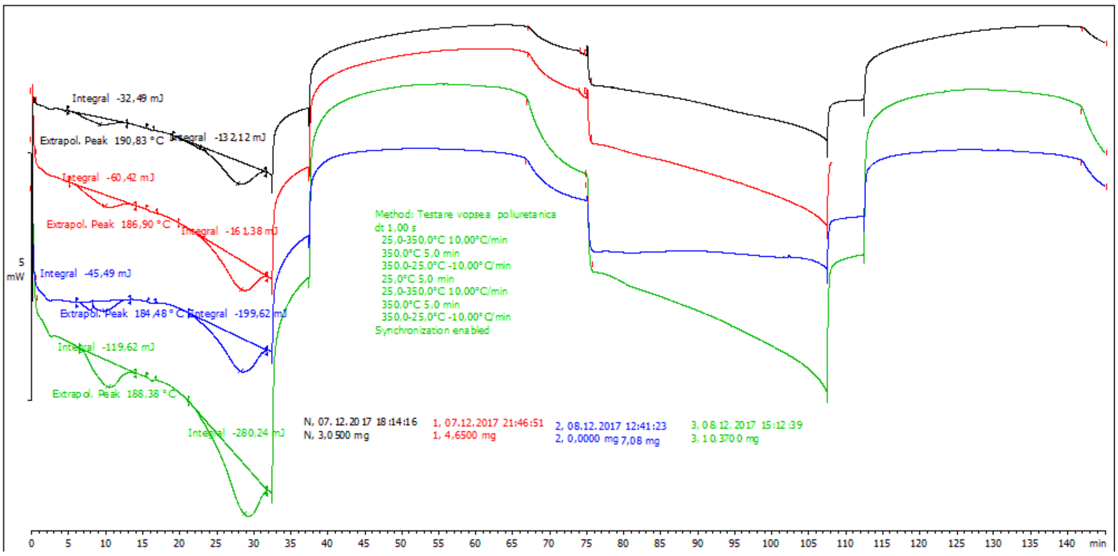

(a)

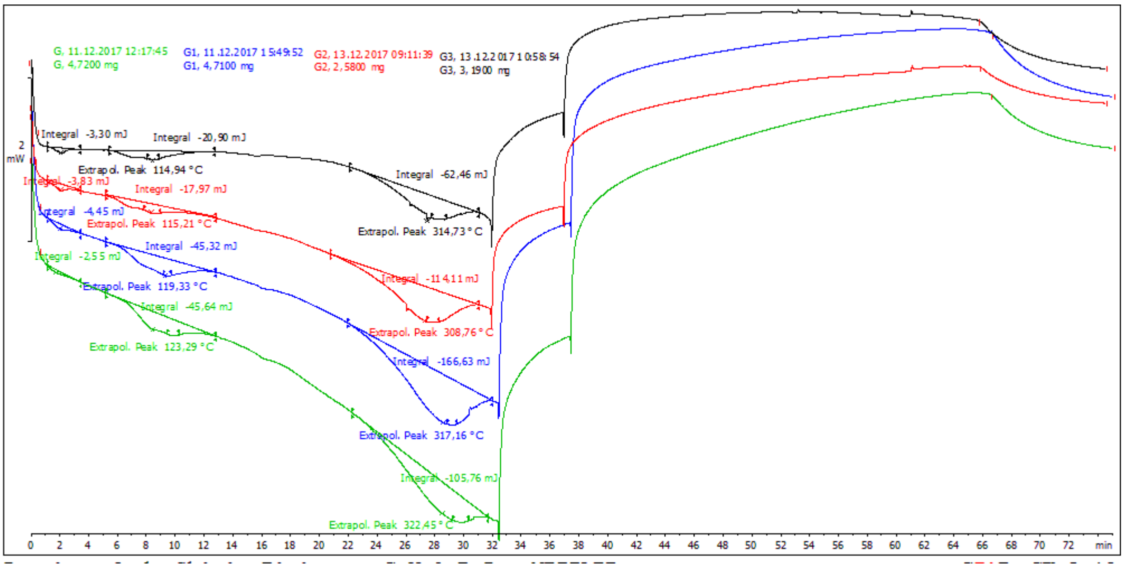

(b)

Figure S6. DSC curves for a) N, N1, N2, N3, and b) G, G1, G2, and G3 samples in nitrogen

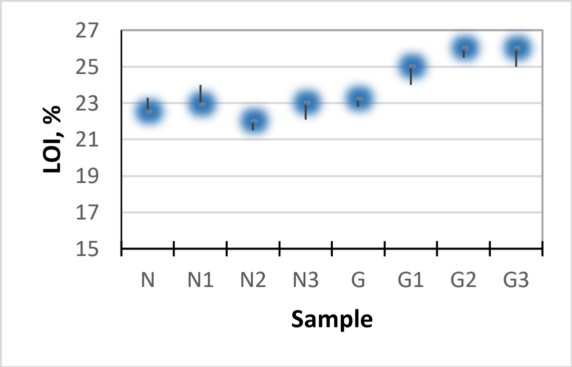

Figure S7. LOI data
